# Supplementary material for: Multiple marginalized identities and internalized HIV stigma among people living with HIV in South Florida: An intersectional approach
Source: PLoS One. 2025 Aug 19;20(8):e0329966. doi: 10.1371/journal.pone.0329966 (PMC12364348; doi:10.1371/journal.pone.0329966)
Supplement: Attachment — Submitted filename: Response to Review.docx [file pone.0329966.s003.docx]

**Response to Review**

*Editor Comments*

Thank you for submitting your manuscript to PLOS ONE. After careful consideration, we feel that it has merit but does not fully meet PLOS ONE’s publication criteria as it currently stands. Therefore, we invite you to submit a revised version of the manuscript that addresses the points raised during the review process.

***Thank you for this helpful feedback. We have addressed these concerns below.***

While the authors have made commendable efforts in addressing the reviewers’ comments, a few minor issues remain, please address the following points:

Reframe language that implies stigma results from individuals' identities, instead, emphasize the structural systems (e.g., racism, sexism) that produce stigma.

***We have further clarified this by adding language to emphasize that stigma results from structural systems of inequity such as racism, sexism, and heterosexism, rather than from individuals’ marginalized identities. Please see an example of the reframed language below that can be found throughout the paper:***

***“Furthermore, PLWH who also identify with other marginalized identities (e.g., race, ethnicity, sexual orientation) may face even more complex patterns of stigma fueled by both misleading narratives surrounding the HIV epidemic and other systemic structures of inequity (e.g., racism, heterosexism, etc.) these individuals are burdened by.”***

Replace references to identities (e.g., race, gender) as social determinants of health (SDOH) with references to the structural forces that shape health outcomes.

***Thank you for this important feedback. We have removed all references to identities as social determinants of health and replaced these with references to the structural systems of inequality that shape health outcomes. Please see the examples below on pages 19 and 21.***

***“Unlike several other of the evaluated marginalized identities, age served as a protective factor against internalized HIV stigma in its interactions with both race and sexual orientation.” (page 19)***

***“Thus, the study makes an important contribution to the literature by identifying intersectional identities and the associated structural systems of inequality that are associated with internalized HIV stigma, informing future research and the development of stigma-reduction interventions to improve both general and HIV-related health outcomes for PLWH.” (page 21)***

Provide a clearer justification for the grouping of diverse racial or gender identity categories (e.g., “non-white” or “non-cis male”).

***Consistent with prior research (Algarin et al., 2019), we chose to dichotomize all five identity variables (race, ethnicity, gender, age, and sexual orientation) to focus the analyses on internalized HIV stigma among marginalized-group identities (as opposed to non-marginalized group identities). A significant reason behind this choice was that breaking down the study sample into more specific intersectional groups to examine sub-group differences would create very small sample sizes for some of the groups (e.g., Asian transgender men), leading to decreased statistical power that would negatively affect our ability to identify significant findings. In addition, this may also create confidentiality issues due to the very small sample sizes for some of the intersectional groups.***

***Further, have also rephrased language throughout the manuscript to more clearly differentiate between the marginalized-group identities and non-marginalized group identities for each of the five identity variables (e.g., “white” and “racial identities other than white”, “heterosexual” and “sexual identity other than heterosexual”, “cisgender man” and “gender identity other than cisgender man”.***

Discuss the potential implications of combining heterogeneous participant groups on the interpretation of findings.

***This is discussed in the limitations section of the manuscript. Please see an example of the language below:***

***“Third, the demographic variables included in the study were dichotomized to reflect a marginalized group and a non-marginalized group. This approach may have created challenges with capturing within-group differences (e.g., the gender marginalized-group identity variable included both cisgender women and transgender individuals; the racial marginalized-group identity variables included individuals who identified as Black/African American, Asian, Native American/Pacific Islander, and other races). Future research with larger sample sizes should more explicitly tease out these differences in sub-groups.”***

Ensure all reviewer comments are explicitly and thoroughly addressed in both the revised manuscript and the response letter. In particular, revisit any areas where concerns were acknowledged but not fully incorporated into the manuscript.

***We have gone through once again, thoroughly, to make sure that all of the concerns have been incorporated into the manuscript, per some of the comments above this has been generally the case. We discovered one area where a concern was acknowledged, but not incorporated into the manuscript: the inclusion of only two-way interactions in the models and not three- or four-way interactions. Upon consultation with colleagues with significant expertise in this space, authors chose not to include additional interactions because with this sample size, three- and four-way interactions would not likely produce meaningful results.***

***As described in our previous revision, in writing the current paper, we hoped to begin to address a gap in the literature - since to our knowledge, there are no prior studies examining marginalized-group identities and internalized HIV stigma from an intersectional perspective. Researchers have historically utilized qualitative methods to examine intersectionality; therefore, ideas regarding the most appropriate quantitative methods to do so are continuing to emerge (Bowleg & Bauer, 2016). While analyzing three- and four-way interactions would be an important next step in truly evaluating intersectionality, we chose to include only two-way interactions in the models due to sample size challenges and the need to protect participant confidentiality. Unfortunately, with these challenges, there was little we could do with three- and four-way interactions in the current study. In addition, it is well-established in the literature that there are limitations to quantifying intersectionality; however, using the multiplicative approach to examine only the interactions, without the main effects in the model, is widely agreed upon to be the most appropriate method for quantitative studies seeking to take an intersectional approach (Bowleg & Bauer, 2016).***
